# Surgical anatomy of the supracarinal esophagus based on a minimally invasive approach: vascular and nervous anatomy and technical steps to resection and lymphadenectomy

**DOI:** 10.1007/s00464-016-5186-1

**Published:** 2016-08-23

**Authors:** Miguel A. Cuesta, Nicole van der Wielen, Teus J. Weijs, Ronald L. A. W. Bleys, Suzanne S. Gisbertz, Peter van Duijvendijk, Richard van Hillegersberg, Jelle P. Ruurda, Mark I. van Berge Henegouwen, Jennifer Straatman, Harushi Osugi, Donald L. van der Peet

**Affiliations:** 1 Department of Surgery, VU Medical Center, De Boelelaan 117, ZH 7F020, 1081 HV Amsterdam, The Netherlands; 2 Department of Surgery, University Medical Center Utrecht, Utrecht, The Netherlands; 3 Department of Anatomy, University Medical Center Utrecht, Utrecht, The Netherlands; 4 Department of Surgery, Academic Medical Center, Amsterdam, The Netherlands; 5 Department of Surgery, Gelre Hospital, Apeldoorn, The Netherlands; 6 Department of Surgery, Osaka University, Osaka, Japan

**Keywords:** Esophagus, Upper mediastinum, Bronchial arteries, Recurrent laryngeal nerve, Lymphadenectomy, Thoracoscopy

## Abstract

**Background:**

During esophageal dissection and lymphadenectomy of the upper mediastinum by thoracoscopy in prone position, we observed a complex anatomy in which we had to resect the esophagus, dissect vessels and nerves, and take down some of these in order to perform a complete lymphadenectomy. In order to improve the quality of the dissection and standardization of the procedure, we describe the surgical anatomy and steps involved in this procedure.

**Methods:**

We retrospectively evaluated twenty consecutive and unedited videos of thoracoscopic esophageal resections. We recorded the vascular anatomy of the supracarinal esophagus, lymph node stations and the steps taken in this procedure. The resulting concept was validated in a prospective study including five patients.

**Results:**

Seventy percent of patients in the retrospective study had one right bronchial artery (RBA) and two left bronchial arteries (LBA). The RBA was divided at both sides of the esophagus in 18 patients, with preservation of one LBA or at least one esophageal branch in all cases. Both recurrent laryngeal nerves were identified in 18 patients. All patients in the prospective study had one RBA and two LBA, and in four patients the RBA was divided at both sides of the esophagus and preserved one of the LBA. Lymphadenectomy was performed of stations 4R, 4L, 2R and 2L, with a median of 11 resected lymph nodes. Both recurrent laryngeal nerves were identified in four patients. In three patients, only the left recurrent nerve could be identified. Two patients showed palsy of the left recurrent laryngeal nerve, and one showed neuropraxia of the left vocal cord.

**Conclusions:**

Knowledge of the surgical anatomy of the upper mediastinum and its anatomical variations is important for standardization of an adequate esophageal resection and paratracheal lymphadenectomy with preservation of any vascularization of the trachea, bronchi and the recurrent laryngeal nerves.

The concept that the aorto-esophageal ligament (also called the meso-esophagus) connects the anterior aspect of the descending aorta to the left side of the esophagus and contains blood vessels, lymph vessels and nerves can only be valid for the infracarinal esophagus [1]. As the supracarinal esophagus is vascularized on both sides, it is of utmost importance to know the variable anatomy when performing an esophageal resection up to the thoracic apex and also for doing a complete lymphadenectomy on both sides of the trachea and of both recurrent laryngeal nerves [2–5].

The manner in which Eastern World (EW) and Western World (WW) surgeons indicate and perform a lymphadenectomy (LN) differs. In the WW, the majority of surgeons only perform a two-field lymphadenectomy, whereas in Japan the three-field lymphadenectomy is the standard treatment for middle and high located squamous cell carcinomas [6–12]. Also, the range of extent for the two-field lymphadenectomy varies. According to the Consensus Conference of the International Society of Diseases of Esophagus (ISDE), held in Munich 1994, three extents of mediastinal lymphadenectomy are found: the standard LN, the extended two-field LN and the so-called total mediastinal LN [13]. Classically, holding for a two- or three-stage esophageal resection for distal esophageal and gastroesophageal junction adenocarcinomas, after the neoadjuvant therapy expert opinions are seen to be divided. There are surgeons who will not routinely resect paratracheal and recurrent laryngeal nerve stations and only resect these if suspect for metastasis, whereas others may routinely perform a paratracheal lymphadenectomy for the most part only on the right side [13–15]. However, during a thoracoscopic approach these stations are clearly visible and resection, if needed, can be performed with optimal safety [16–18]. There is some discussion about how to dissect this area and which structures have to be dissected and divided in order to perform an adequate resection with optimal functional outcome for neighbor organs, especially tracheobronchial tree and lung function [19–21]. Observations of the supracarinal esophagus during thoracoscopic dissection in prone position prompted us to carry out a retrospective and prospective study with in vivo imaging. And additionally to describe the surgical anatomy concerning anatomical structures and planes of this important part of the upper mediastinum, and doing this in order to perform an adequate esophageal resection with an adequate lymphadenectomy.

## Materials and methods

### Retrospective study

The surgical anatomy of the supracarinal esophagus and the required steps for resection and lymph node dissection were studied by one of the surgeons (MAC) in 20 consecutive unedited videos of patients in whom a total mediastinal lymphadenectomy had been performed between January 2012 and February 2014. Indication for total mediastinal LN included all patients with squamous cell cancer (SCC) and esophageal cancer in distal locations (mostly located in the middle or high esophagus) with suspected involvement of the lymph nodes in the high mediastinum, as determined by PET-CT scan. All patients received neoadjuvant chemoradiotherapy according to the CROSS scheme [22].

### Prospective study

A verification study was performed in five consecutive patients in two hospitals between November 2014 and March 2015, four patients with SCC and one patient with an adenocarcinoma (AC) located in the mid-lower esophagus with suspicion of involvement of the lymph nodes in the upper mediastinum. All patients received neoadjuvant chemoradiotherapy according to the CROSS scheme [22].

### Operative technique by thoracoscopy in prone position

**Fig. 1:**
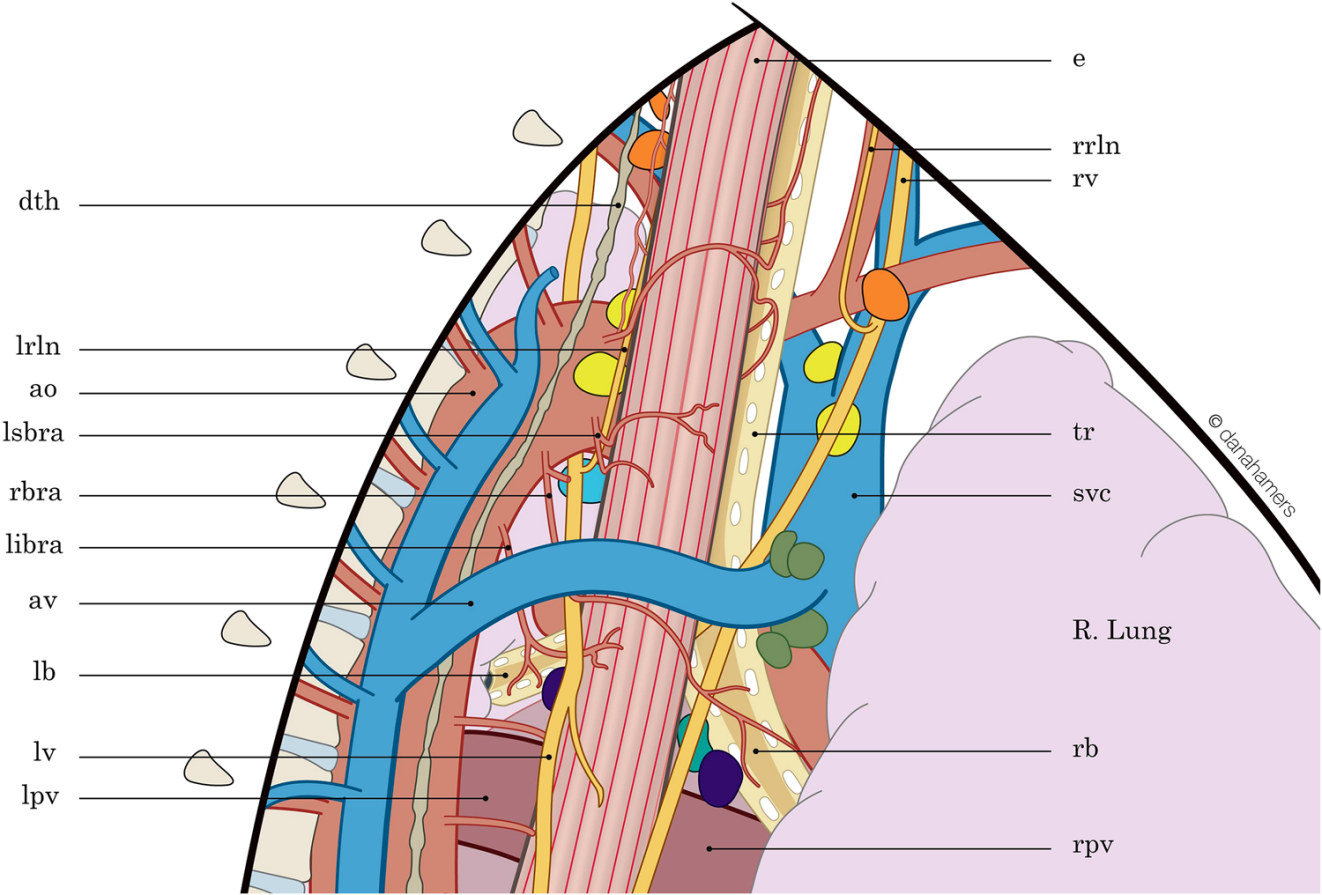
Surgical anatomy of the supracarinal mediastinum. View in prone position

**Fig. 2:**
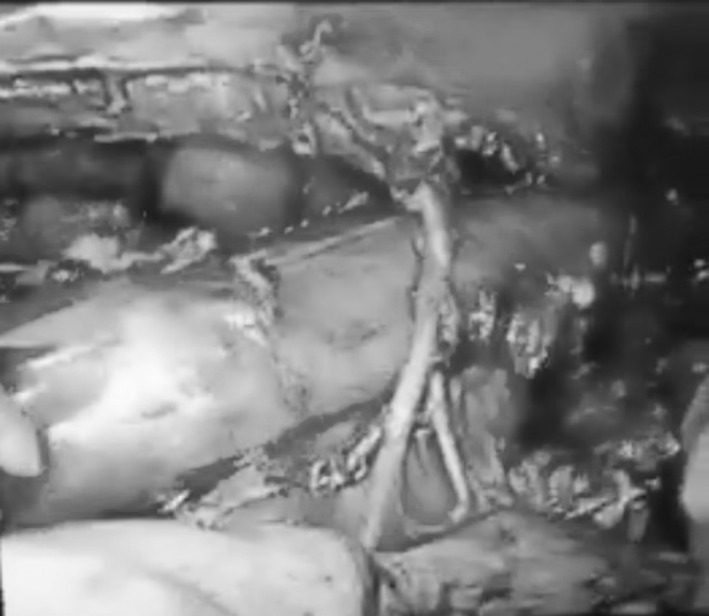
Right bronchial artery

**Fig. 3:**
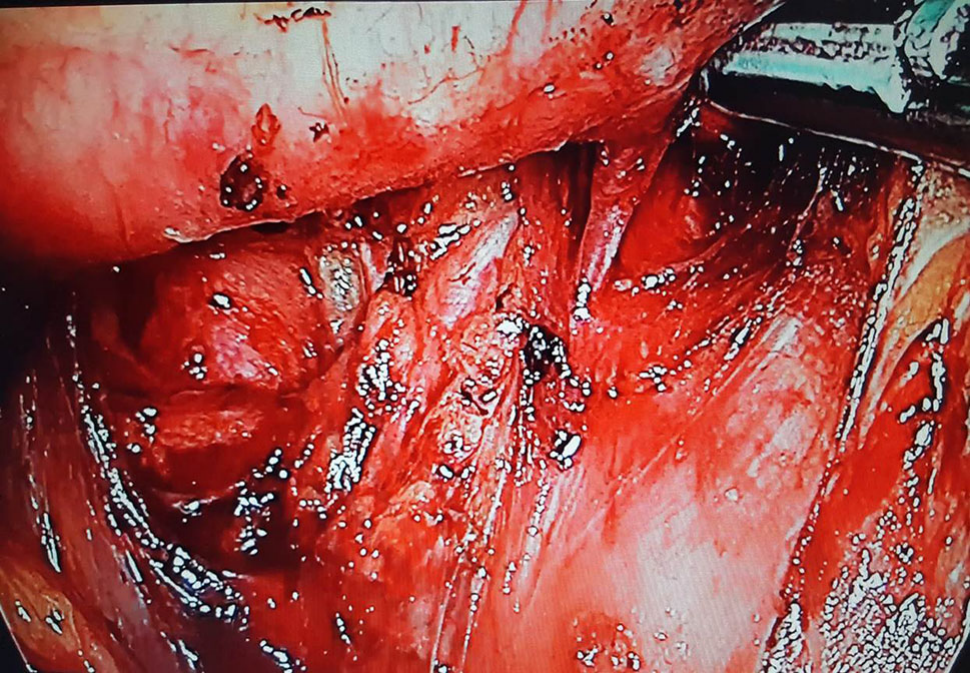
Left bronchial branch to the trachea

In order to perform minimally invasive esophagectomy (MIE) by thoracoscopy in prone position, we started by opening the mediastinal pleura on both sides of the esophagus, along the right lung and on the left side along the azygos vein. Furthermore, the upper mediastinal pleura is opened on the right side between the vagal nerve and the superior vena cava and on the left side along the left border of the trachea up to the thoracic apex (Fig. 1). After this, the azygos vein is divided by means of a vascular stapler and the right vagal nerve is divided at the lower level of the right bronchus. The next step is to dissect the esophagus with the periesophageal lymph nodes free from the pars membranacea of the trachea and the vascular lateral attachments. Arterial supply of the supracarinal esophagus comes from both sides of the esophagus, and the bronchial arteries also supply the trachea and both bronchi. There is much variation in the numbers and patterns of the bronchial arteries, the most frequent (70 %) being two on the left side and one on the right, but all combinations of one or two on each side are possible. Because of this variability, it is necessary to identify the bronchial arteries during surgery—that is between left and right—before dividing them. The right bronchial artery (RBA) is a branch of the third posterior intercostal artery, and the left bronchial arteries (LBA) are coming directly from the aortic arch. According to the anatomy, the artery that supplies blood to the right bronchus is identified as the RBA (Fig. 2) and should be divided. On the left side, there are frequently two LBA possible to identify during the esophageal dissection from the aorta arch. If we pull the esophagus, the tissue going to esophagus from the aorta is divided, and frequently one of the LBA can be identified. But identifying the left bronchus and dissecting the esophagus free from it, vessels coming from the aortic arch to the left bronchus can be preserved. In practice, from distal to proximal, this implies to divide the RBA at both sides of the esophagus and on the left side to divide the vessels between the aortic arch and the esophagus (one or both LBA), trying to preserve if possible one branch going to the left bronchus (Fig. 3).

**Fig. 4:**
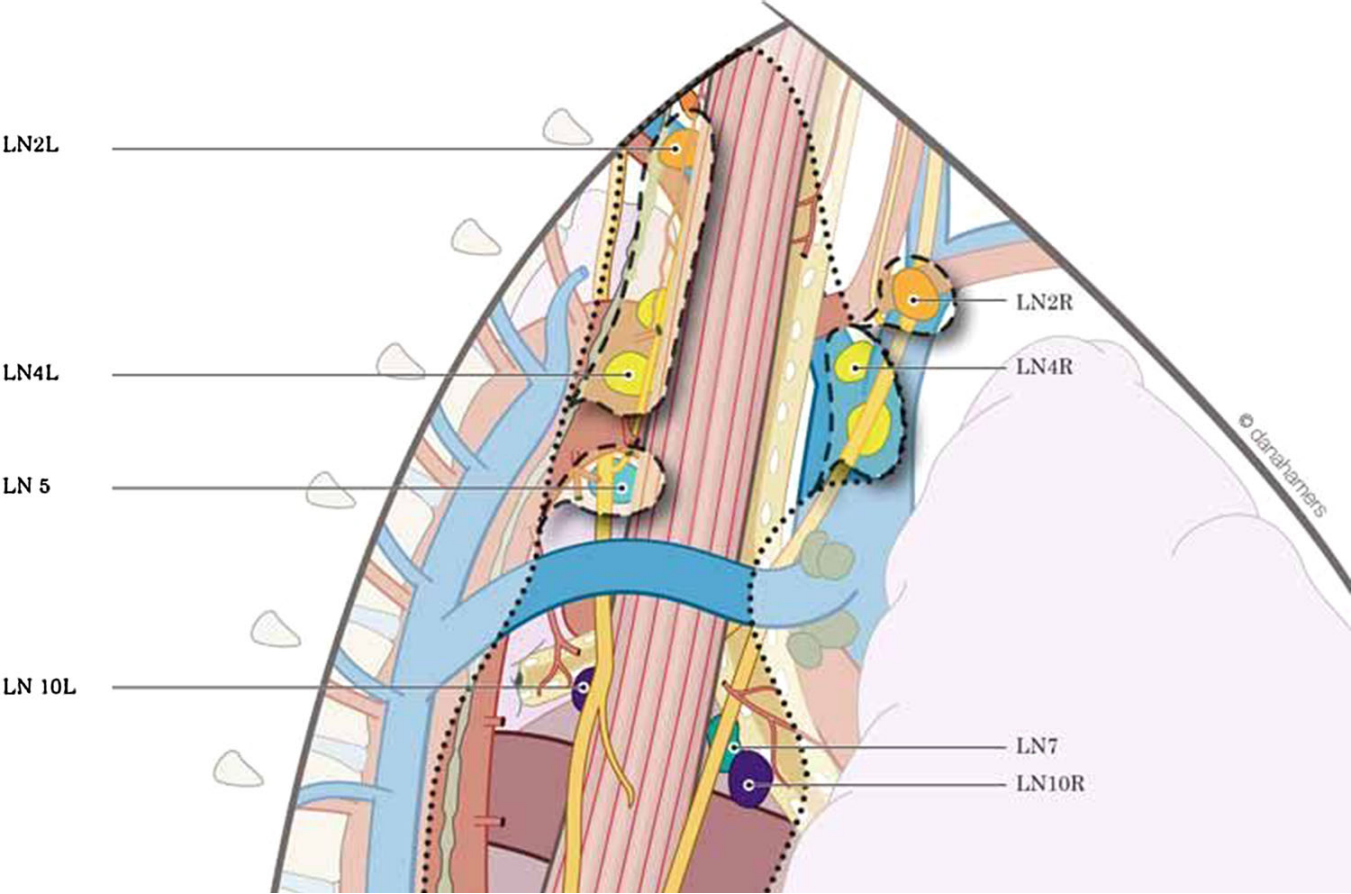
Lymphadenectomy groups in supracarinal mediastinum, station 2 and 4L and R and station 5. View in prone position

Furthermore, these bronchial arteries are not terminal arteries, having many anastomoses in front of the tracheobronchial tree (Fig. 4).

The right vagal nerve goes from lateral behind the subclavian artery, where the right recurrent laryngeal nerve recurs, and courses laterally of the trachea to cross posteriorly of the main right bronchus, giving numerous branches to the right lung and going to the esophagus as the esophageal plexus. The left vagal nerve goes posteriorly of the aortic arch, where the left recurrent nerve recurs (ascending along the left side of the trachea), and from there crosses to the posterior aspect of the main left bronchus and, before access to the esophagus, gives numerous branches to the left lung [21].

**Fig. 5:**
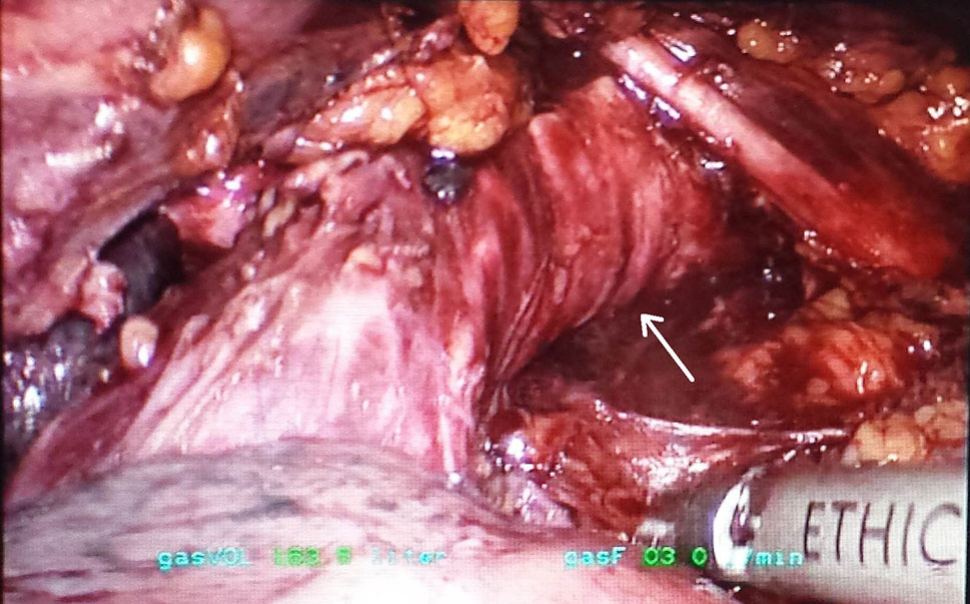
Gentle retraction of the trachea in order to perform left paratracheal lymphadenectomy (*white arrow* shows the trachea)

**Fig. 6:**
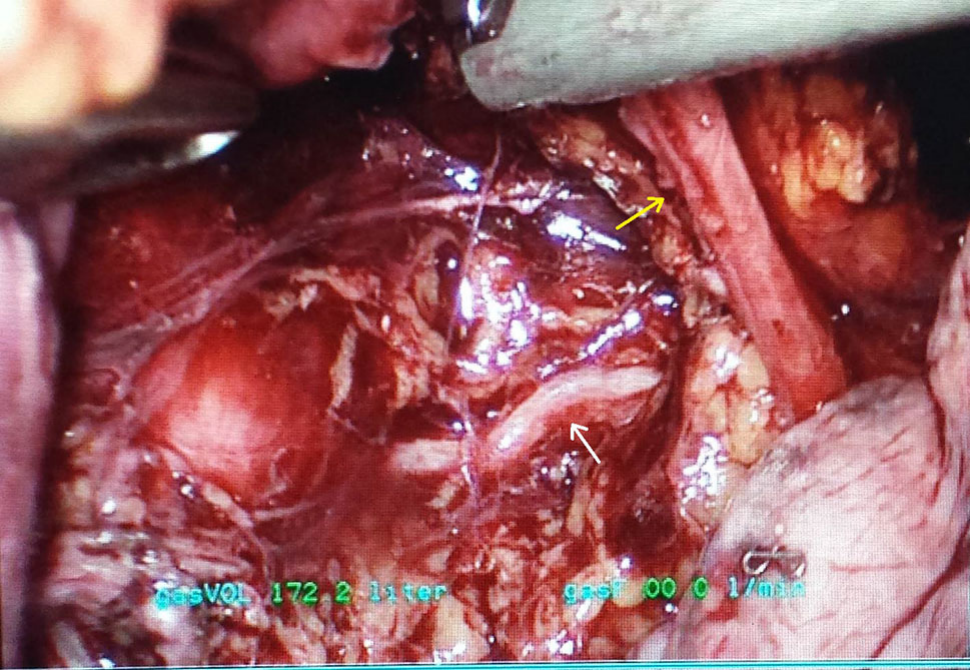
Identification of left recurrent laryngeal nerve gently retracting the trachea from right side (*white arrow* shows the left laryngeal nerve, and *yellow arrow* shows the trachea) (Color figure online)

If total mediastinal LN is indicated, the high paratracheal mediastinal lymphadenectomy is performed on both sides (Fig. 3), and on the right side the field may be—for practical purposes—divided into two areas: one from the superior vena cava to the right vagal nerve and the other from the right vagal nerve to the trachea. During dissection on the right side, LN of stations 4R and 2R should be performed after identification of the right recurrent laryngeal nerve at the level of the right subclavian artery. On the left side, after LN of the subaortic station (station 5), dissection of station 2L and 4 L can be performed in two ways, frontally along the trachea after identification of the left recurrent laryngeal nerve or to do “en bloc” LN starting with the right side, continuing with a gentle retraction of the trachea through its anterior aspect (in prone position, Fig. 5) to finally end on the left side with identification and preservation of the left recurrent laryngeal nerve (Fig. 6).

### Lymph node classifications

There are two lymph node grading classifications specific for esophageal cancer including both mediastinal and abdominal lymph nodes stations: the Japanese Society Esophageal Disease (JSED) and the American Joint Committee on Cancer (AJCC). Moreover, there is the lymph node grading of the International Association Study Lung Cancer (IASLC) specific for mediastinal lymph node stations [13, 23, 24].

The infracarinal, bronchial and periesophageal lymph nodes can be retrieved en bloc with the specimen, whereas the rest will be taken out separately after resection.

## Results

The patient characteristics of the retrospective and prospective study are depicted in Table 1.

**Table 1:** Characteristics and outcomes of the study groups

	Retrospective (*n* = 20)	Prospective (*n* = 5)
Histology		
Adenocarcinoma	2 (10 %)	1 (20 %)
Squamous cell carcinoma	18 (90 %)	4 (80 %)
Vascular anatomy		
1 RBA + 2 LBA	14 (70 %)	5 (100 %)
1 RBA + 1 LBA	6 (30 %)	0
Preserved RBA	2	1
Preserved 1 LBA	14	0
Preserved 1 LBA to trachea	6	5
Lymphadenectomy		
Separate	20	2
En bloc	0	3
Visualization recurrent laryngeal nerve		
Left recurrent laryngeal nerve	2	1
Both recurrent laryngeal nerves	18	4
Complications		
Respiratory infection	2	1
Anastomotic leakage	2	1
Recurrent nerve palsy	2	1

Eighteen patients had middle and high located SCC, and two patients had AC with suspected involvement of the lymph nodes in the upper mediastinum, as determined by PET-CT scan.

### Anatomical findings

In all patients, we identified arterial and nervous structures in the course of the paratracheal lymphadenectomy (2L + R and 4L + R). Anatomical findings are depicted in Table 1.

Fourteen out of 20 patients had one right bronchial artery and two left bronchial arteries (70 %), whereas the other six patients had one right and one left bronchial artery.

In two out of twenty patients in the *retrospective* study, the RBA was preserved. In the other eighteen patients, the artery was divided during dissection at both sides of the esophagus. In fourteen patients, at least one LBA was preserved on the left side, and in six others the branches to the trachea and left bronchus of the superior LBA could be preserved.

Regarding the *prospective* study, in all these five cases one right bronchial artery and two left arteries were found, with at least one branch of the superior LBA to the trachea and left bronchus being preserved. Moreover, in one patient the RBA was preserved and in the other four patients the artery was divided at both sides of the esophagus.

Moreover, in eighteen out of twenty patients in the *retrospective* study, the right and left recurrent laryngeal nerves were visualized, whereas in two only the left was seen while in the *prospective* study the visualization on the left recurrent laryngeal nerve was reached in all patients, and on the right side only in four could be visualized with the fifth patient having an aberrant right subclavian artery (subclavian lusoria).

In all patients in the *retrospective* study, the upper mediastinal LN was performed separately, first right and then left along the left recurrent laryngeal nerve. While in the *prospective* study, in two patients left and right were done separately, whereas in the other three it was performed en bloc.

### Oncological outcome

The median number of resected lymph nodes in the supracarinal area was 11 (range 8–14). In six patients (five in the retrospective and one in the prospective study), a median of three lymph node metastases (range 2–5) was found. R0 resection was achieved in 95 % of the patients in the retrospective and in 100 % of the prospective study.

### Complications

In the retrospective study, two patients developed a postoperative lung infection, two had a palsy of the left recurrent nerve, and in two others a cervical anastomosis leakage was observed. In the prospective study, there was one patient with respiratory infection, another had a neuropraxia of the left vocal cord, and one had an anastomotic leakage.

## Discussion

Having a comprehensive concept of the live surgical anatomy is necessary for ensuring the anatomical accuracy as well as reproducibility of radical resections for cancer.

During conventional transthoracic esophagectomy for cancer, the upper mediastinal area may be a difficult area to visualize, to comprehend and frequently so to dissect for the purpose of performing an adequate esophageal resection and lymphadenectomy. It is a complex area in which the mediastinal vital structures move up in direction to the head and superior extremities. In the thoracoscopic approach, however, precise dissection of this part of the anatomy is possible by the augmentation and the precision of the imaging obtained [18, 25–27].

Moreover, when performing an upper mediastinal lymphadenectomy, indications for it and even its extension are still not well established; moreover, its terminology (classification) is not completely standardized [6–15, 23, 24, 28].

Minimally invasive esophagectomy is increasingly performed [25–27], and definition of surgical anatomy is important to accomplish. In a previous study, we have described and called meso-esophagus the fascia and structures between the aorta and the infracarinal esophagus [1]. In this retrospective and prospective study of minimally invasive esophagectomy (MIE) in prone position, our aim was to describe the surgical anatomy of the upper mediastinum, and the landmarks and structures that have to be divided, in order to perform an adequate esophagectomy and lymphadenectomy. The numbers of complications recorded as a consequence of this dissection, especially palsy of the recurrent laryngeal nerves, can be considered high and may have important consequences for the numbers of respiratory infections [29, 30].

This resulted in a technical annotation on how to perform this challenging dissection in a safe way with the purpose of decreasing the morbidity.

In order to perform the MIE in prone position, first the dissection of the esophagus, periesophageal tissues and subcarinal lymph nodes is performed, which is followed by the dissection of the upper mediastinum. Dissection starts after the opening of the pleura on both sides with division of the vascular structures necessary to free the esophagus and is followed by careful dissection of the esophagus from the pars membranacea of the trachea. After this, if indicated, extended or total lymphadenectomy on the right side is performed followed by lymphadenectomy on the left side.

Bronchial arteries are considered an important vascular and nutritional supply of the esophagus, trachea, bronchi and lungs, but their anatomy shows a great variability. Variations in arterial supply are frequent described but also its terminology for example, high on the right side arterial branches coming from the neck connects with what is called by some esophageal surgeons the tracheoesophageal artery, while by pulmonary surgeons it is called the upper bronchial artery. It originates from the right subclavian artery, runs ventrocaudally and sends branches to trachea and esophagus.

During the operation, it should be carefully observed which vessels are going to the right and which are going to the left side of the esophagus. The finding in our study that 70 % of the patients had one right bronchial artery and two left bronchial arteries is in concordance with the literature [2–6]. Arterial supply of the esophagus, trachea and principal bronchi is common; therefore, it is important to know which arteries should be divided and which preserved during dissection. Important is to preserve as much as possible of the arterial supply to the trachea and bronchi. On the left side, at least one of the two branches should be preserved, with a preference for the superior bronchial artery with only branches to the trachea and bronchus. If only one exists, only the branches to the esophagus should be divided. Failure to do so may result in impairment of the postoperative respiratory function [19, 20]. In this study, we had been able to preserve at least one bronchial artery or the branch of one going to the trachea in all patients. This is important because these bronchial arteries are not terminal arteries, having many anastomoses in front of the tracheobronchial tree.

In this study, the lymphadenectomy was performed separately, upper paratracheal (2R and 2L) and lower paratracheal lymph node stations (4R and 4L) in all patients in the retrospective study and in two patients in the prospective study, while in three patients in the prospective study this was done en bloc. This is a new approach to perform this type of lymphadenectomy and may be performed because of the visibility obtained during thoracoscopy. The majority of the Japanese surgeons perform a three-field lymphadenectomy for middle and high in the esophagus located SCC, whereas the common procedure in the WW is to perform a two-field LN. In Japan, surgeons initiated the intervention with bilateral neck dissection, with control of both recurrent laryngeal nerves. During lymphadenectomy, preservation of the recurrent nerves is essential and careful dissection, especially on the left side, is important in avoiding a traumatic dissection and traction.

No damage of the recurrent laryngeal nerves was seen during the operations in this series, but clinically two palsies and one temporary neuropraxia of the left nerve were observed. Moreover, the numbers of pulmonary infections and anastomotic leakages in the neck are according to the observed in the TIME trial [25].

The use of neoadjuvant chemoradiotherapy does not harm the dissection, and the lymph nodes numbers obtained in this area are considered adequate even if the neoadjuvant chemoradiotherapy is known to reduce the numbers of total lymph nodes removed, possibly having more prognostic than therapeutic value [22, 31].

Questions arise whether the prone position of the patient will generate all these advantages in comparison with the thoracoscopy in lateral position. Kawakubo et al. [32] describe the hybrid semiprone position adopted in some centers in Japan to dissect the paratracheal lymph nodes correctly. They described this position as optimal because the lateral decubitus and prone positions can be achieved by rotating the surgical table. They found that if the prone position is adequate for dissection of the middle and lower mediastinum, then the lateral position is better for the upper mediastinum, especially around the left recurrent laryngeal nerve.

Moreover, to perform the thoracic phase of the robot-assisted esophageal resection (RAMIE) the patient is also positioned from the decubitus into this semiprone position in order to work optimally with the robot and at the same time visualize correctly the upper part of the mediastinum [33].

The pertaining comprehensive description is significant. First, it defines the anatomical structures and landmarks for the optimal esophageal resection and lymphadenectomy if indication exists for this on the upper mediastinum. Second, it describes the steps to operate, pointing out how important it is to divide some vascular structures and preserving others. Third, it describes the two possibilities for lymphadenectomy, en bloc or separate left from right. Another important aspects of this esophageal anatomy may be the description of the vagal nerves and the possible importance of preserving the pulmonary branches to protect the pulmonary function postoperatively [21].

Discussion is still going on about the extension of the lymphadenectomy. Independent of the prognosis, when the lymph nodes in the upper mediastinum are involved, total mediastinal lymphadenectomy is indicated in resectable SCC and AC in any location. In the case of AC, mostly located in the distal esophagus and GEJ with no suspected lymph nodes, and especially after neoadjuvant therapy, the question remains open. Currently, it is the personal choice of the surgeon whether to perform standard, extended or total lymphadenectomy. A lymph node and lymphadenectomy survey the TIGER survey is already performed in order to solve this question, especially in the era of neoadjuvant therapy [34].

Dissection by minimally invasive surgery of the upper mediastinum in esophageal surgery can be demanding, partly because of anatomical variation. Having knowledge of the surgical anatomy and landmarks of the resection will improve an understanding of the anatomy, thereby leading to a more adequate and reproducible surgical resection.

In our study, we have determined that important advantages as obtained by minimally invasive surgery—such as visualization and augmentation—have permitted a more complete knowledge and standardization of the surgical anatomy of the upper mediastinum.
